# Study of the Effect of Methyl Eugenol on Gastric Damage Produced by Spinal Cord Injury Model in the Rat

**DOI:** 10.3390/molecules30010086

**Published:** 2024-12-29

**Authors:** Leticia Cruz-Antonio, María Elena Sánchez-Mendoza, Jazmín García-Machorro, Yaraset López-Lorenzo, Jesús Arrieta

**Affiliations:** 1 Facultad de Estudios Superiores Zaragoza, Universidad Nacional Autónoma de México (UNAM), Av. Guelatao No. 66, Colonia Ejército de Oriente, Iztapalapa, Ciudad de México 09230, Mexico; letycruza@yahoo.com.mx; 2 Laboratorio de Farmacología de Plantas Medicinales Mexicanas, Escuela Superior de Medicina, Instituto Politécnico Nacional, Plan de San Luis y Díaz Mirón, Colonia Casco de Santo Tomás, Miguel Hidalgo, Ciudad de México 11340, Mexico; mesmendoza@hotmail.com (M.E.S.-M.); yarlop_2310@outlook.com (Y.L.-L.); 3 Laboratorio de Medicina de la Conservación, Escuela Superior de Medicina, Instituto Politécnico Nacional, Plan de San Luis y Díaz Mirón, Colonia Casco de Santo Tomás, Miguel Hidalgo, Ciudad de México 11340, Mexico; jazzgama81@gmail.com

**Keywords:** gastroprotection, spinal cord injury, methyl eugenol

## Abstract

Traumatic spinal cord injury (SCI) is a serious medical condition that places patients at high risk of developing gastric ulceration and gastrointestinal bleeding. One preventative strategy involves the use of omeprazole; however, its chronic use is associated with adverse effects, highlighting the need for alternative therapies. This study evaluated the protective effects of methyl eugenol (ME) on gastric mucosal damage in a rat model of SCI. ME was administered orally at doses of 30, 100, and 177 mg/kg in SCI induced at the T9 level, alongside diclofenac or ketorolac (30 mg/kg each). The enzymatic activity of superoxide dismutase, catalase, and glutathione peroxidase was assessed, and the levels of total glutathione and malondialdehyde were determined using biochemical kits. Additionally, stomach histological sections were analyzed. ME exhibited dose-dependent gastroprotective effects, with maximal protection observed at 177 mg/kg in the presence of diclofenac (9.78 ± 2.16 mm^2^) or ketorolac (12.49 ± 2.17 mm^2^). A histological analysis confirmed these findings. In conclusion, methyl eugenol protects the gastric mucosa from SCI-induced damage, with glutathione peroxidase and catalase playing key roles in its mechanism of gastroprotection.

## 1. Introduction

Spinal cord injury (SCI) has a significant impact in terms of mortality and morbidity [1]. Many incidents can lead to SCI, with the main causes being falls and road traffic accidents. In 2016, the annual incidence of SCI in the United States was 54 cases per million people, with a prevalence of 721 to 906 per million people [2]. SCI presents a burden on healthcare systems due to the costly and complex medical support required by patients considering the need for long-term care in addition to the economic consequences of lost productivity [1,3]. Patients with SCI may experience common long-term secondary complications, including respiratory, cardiovascular, urinary, and intestinal issues, spasticity, chronic nociceptive pain, pressure ulcers, osteoporosis, and bone fractures. Therefore, prevention, early diagnosis, and treatment of these complications are essential to limit their progression and improve patients’ health-related quality of life [4].

Another complication in patients with SCI may be acute gastrointestinal ulcerations and bleeding. Several factors may contribute to the occurrence of these complications, such as the use of thrombolytic drugs, anticoagulants, and non-steroidal anti-inflammatory drugs (NSAIDs). However, gastrointestinal ulcerations and bleeding have not received adequate attention, as evidenced by the fact that they are rarely mentioned in the literature on SCI complications.

Chronic nociceptive pain (musculoskeletal or visceral) is common in patients with SCI and is often treated with NSAIDs [5]. Older-generation NSAIDs are known to cause gastric ulcers by non-selectively inhibiting COX [6], which worsens the condition in individuals with SCI. Furthermore, patients with acute quadriplegia due to trauma are more likely to develop stress gastric ulceration and subsequent bleeding [7]. Stress gastric ulcers are treated with antacids or H_2_ antagonists yet still occur despite treatment [8]. In tetraplegic patients, treatment is particularly difficult since these patients have a higher frequency of deep vein thrombosis, and the treatments of both problems are diametrically opposed. Additionally, they appear to be resistant to H_2_ antagonists and antacids [8].

Omeprazole, a proton pump inhibitor, is another drug that is widely used to treat gastric ulcers and has been shown to be effective in treating gastric stress ulcers in rats with spinal cord section. However, prolonged use of these inhibitors in clinical studies has been associated with irreversible adverse effects, such as decreased absorption of vitamin B12 (which can lead to dementia, neurological damage, and anemia), hypergastrinemia [9], acute myocardial infarction [10], pancreatic cancer [11], gastric neoplasia (stomach cancer), kidney diseases, bone fractures, impaired absorption of micronutrients, liver disease, and an increased risk of infections [12].

This highlights the need for new therapeutic alternatives with minimal adverse effects. Methyl eugenol is one such compound, and its gastroprotective activity has been demonstrated in a model of gastric lesions induced by ethanol. Therefore, the objective of the present study is to determine whether methyl eugenol protects the gastric mucosa from gastric damage in a rat spinal cord injury model in the presence of diclofenac and ketorolac.

## 2. Results

### 2.1. Gastroprotective Activity

**Figure 1:**
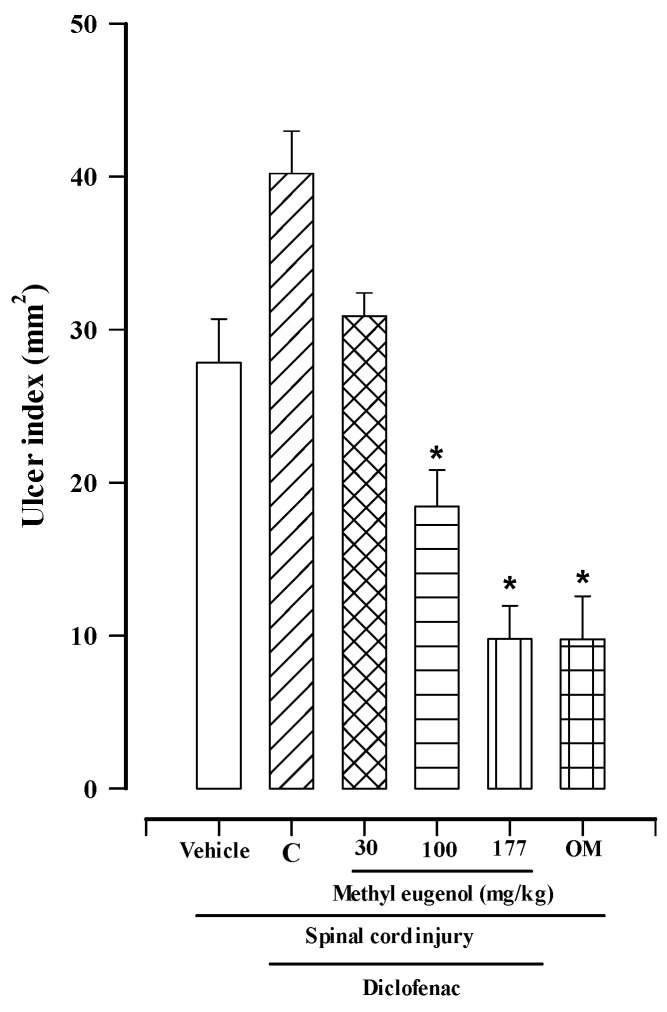
Gastroprotective effects of methyl eugenol on gastric lesions induced by SCI in rats with diclofenac. Bars represent the mean ± SEM (*n* = 8). * *p* < 0.05 vs. vehicle based on the Kruskal–Wallis test followed by Dunn’s multiple comparisons test. C = diclofenac control group; OM = omeprazole.

**Figure 2:**
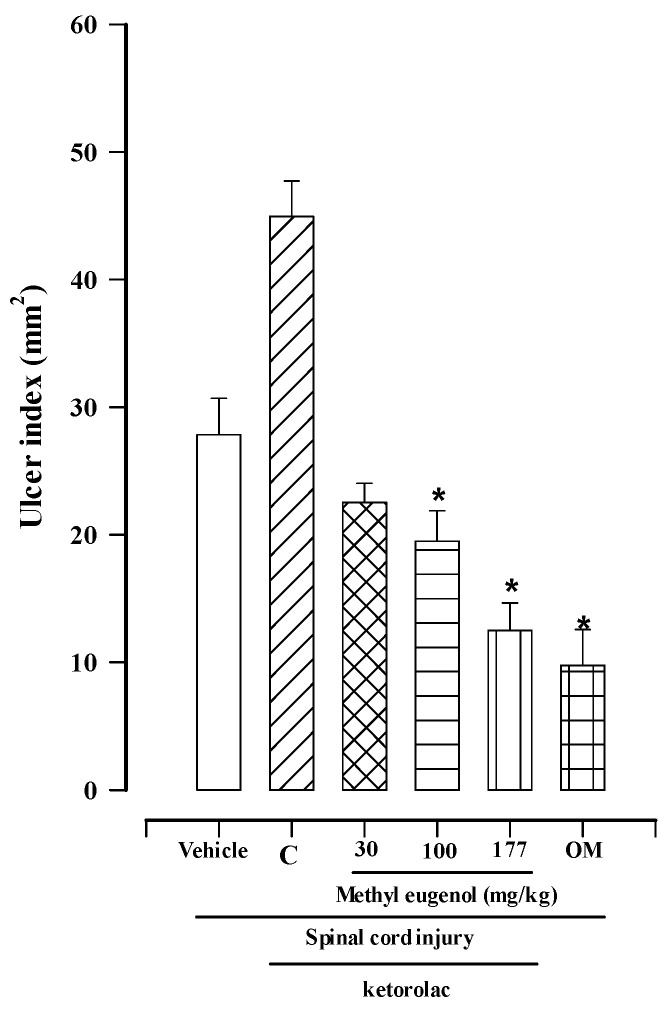
Gastroprotective effects of methyl eugenol on gastric lesions induced by SCI and ketorolac. Bars represent the mean ± SEM (*n* = 8). * *p* < 0.05 vs. vehicle based on the Kruskal–Wallis test followed by Dunn’s multiple comparisons test. C = ketorolac control group; OM = omeprazole.

The results of the evaluation of the gastroprotective activity of methyl eugenol in the SCI model at the T9 level in rats, in the presence of diclofenac or ketorolac, are shown in Figure 1 and Figure 2.

Figure 1 shows the results of SCI plus diclofenac. The administration of diclofenac (30 mg/kg, control group) caused greater gastric damage compared to the vehicle control group (40.22 ± 2.76 mm^2^ and 27.84 ± 2.87 mm^2^, respectively). Methyl eugenol showed a dose-dependent protective effect in the SCI plus diclofenac model, with the maximum effect observed at a dose of 177 mg/kg, followed by doses of 100 and 30 mg/kg (9.78 ± 2.16 mm^2^, 18.43 ± 2.39 mm^2^, and 30.87 ± 1.52 mm^2^, respectively). The ulcer index obtained with a dose of 177 mg/kg was similar to that of omeprazole at 40 mg/kg (9.76 ± 2.81 mm^2^).

Figure 2 shows the results of SCI plus ketorolac. The vehicle control group produced an ulcer index of 27.84 ± 2.87 mm^2^. The presence of ketorolac (30 mg/kg) worsened the gastric damage caused by SCI, resulting in an ulcer index of 44.95 ± 2.77 mm^2^. Methyl eugenol demonstrated a dose-dependent effect in the SCI plus ketorolac model, with maximum gastroprotection at a dose of 177 mg/kg (12.49 ± 2.17 mm^2^), followed by doses of 100 and 30 mg/kg (19.48 ± 2.38 mm^2^ and 22.51 ± 1.54 mm^2^, respectively). The ulcer index after omeprazole administration (40 mg/kg) was 9.76 ± 2.28 mm^2^, a value not significantly different from that obtained with 177 mg/kg of methyl eugenol (Figure 2).

### 2.2. Antioxidant Activity

**Figure 3:**
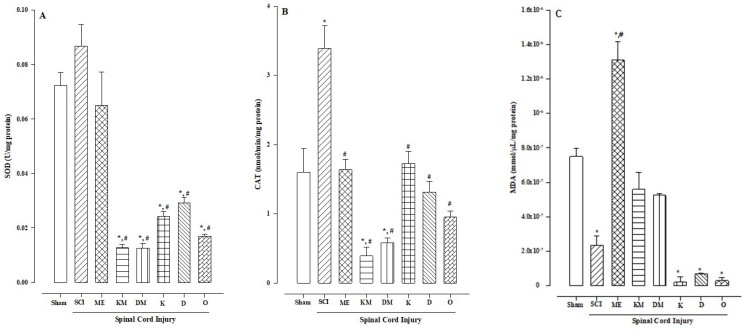
Effects of the antioxidant activity of methyl eugenol (177 mg/kg) on gastric lesions induced by spinal cord injury (SCI) plus diclofenac or ketorolac. (**A**) Determination of superoxide dismutase activity (SOD). (**B**) Results of catalase activity (CAT). (**C**) Determination of malondialdehyde (MDA). Abbreviations: ME = methyl eugenol; KM = ketorolac plus methyl eugenol; DM = diclofenac plus methyl eugenol; K = ketorolac; D = diclofenac; O = omeprazole. Bars represent the mean ± SEM (n = 8). * *p* < 0.05 vs. sham group; # *p* < 0.05 vs. SCI group based on the one–way analysis of variance (ANOVA) followed by Dunnett’s test.

The superoxide dismutase (SOD) enzyme increased in the SCI group without a significant difference compared to the sham group treated with methyl eugenol. The groups treated with ketorolac and diclofenac, alone or in combination with methyl eugenol, showed reduced SOD activity (Figure 3A), with a significant difference (*p* < 0.0005) compared to the sham and SCI groups. This result was also observed in the control group treated with omeprazole (reference drug).

The catalase (CAT) enzyme increased in the SCI group compared to the sham group (Figure 3B), with a significant difference (*p* < 0.0005). In the groups treated with methyl eugenol, ketorolac, diclofenac, and omeprazole, the CAT levels were similar to those in the sham group (no significant difference). However, in the groups treated with the combination of methyl eugenol plus ketorolac and methyl eugenol plus diclofenac, the CAT activity decreased, with significant differences compared to both the sham and SCI groups (*p* < 0.009 and *p* < 0.0005, respectively).

In the sham group, the malondialdehyde (MDA) levels were found at concentrations of 7.49 × 10^−7^ mmol/μL/mg of protein, with a significant difference in the SCI, diclofenac, ketorolac, and omeprazole groups, where the MDA levels were reduced to ≤2.34 × 10^−7^ mmol/μL/mg of protein (Figure 3C). In contrast, in the group treated with methyl eugenol, the MDA levels increased to 1.31 × 10^−6^ mmol/μL/mg of protein, with a significant difference compared to both the sham and SCI groups (*p* < 0.0005).

**Figure 4:**
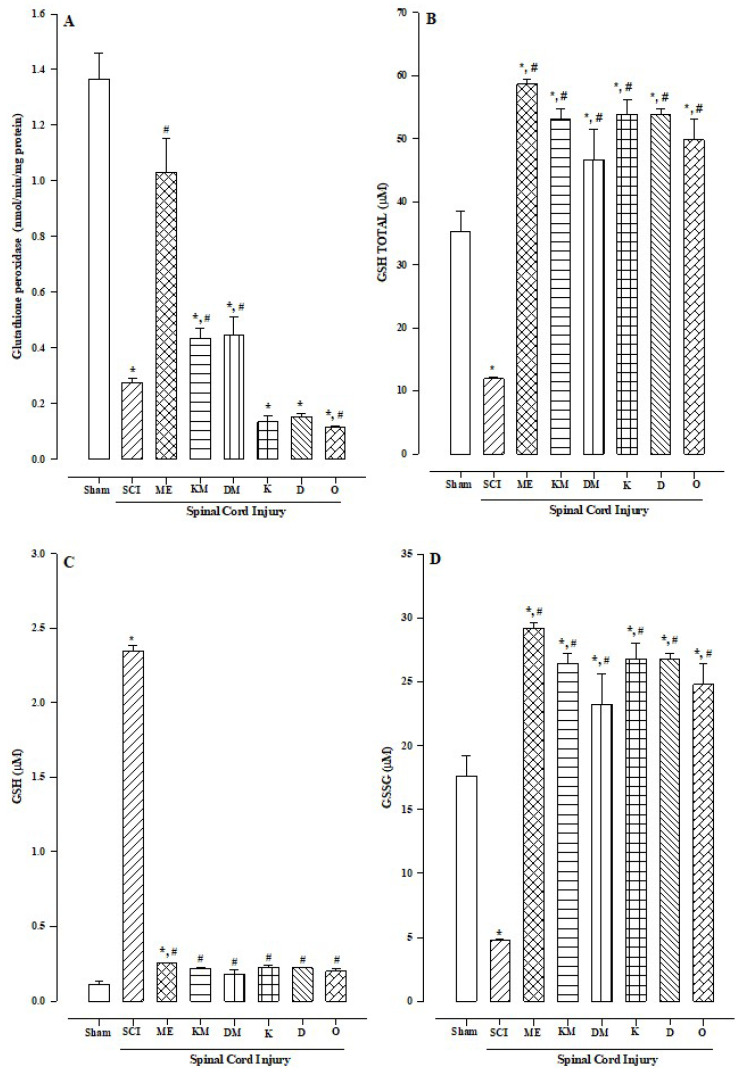
Effects of the antioxidant activity of methyl eugenol (177 mg/kg) on glutathione contained in gastric tissue. Gastric lesions were induced by spinal cord injury (SCI) plus diclofenac or ketorolac. (**A**) Determination of the glutathione peroxidase. (**B**) Total glutathione (GSH total). (**C**) Reduced glutathione (GSH). (**D**) Oxidized glutathione (GSSG). Abbreviations: ME = methyl eugenol; KM = ketorolac plus methyl eugenol; DM = diclofenac plus methyl eugenol; K = ketorolac; D = diclofenac; O = omeprazole. Bars represent the mean ± SEM (n = 8). * *p* < 0.05 vs. sham group; # *p* < 0.05 vs. SCI group based on the one–way analysis of variance (ANOVA) followed by Dunnett’s test. Effects of the antioxidant activity of methyl eugenol (177 mg/kg) on glutathione contained in gastric tissue. Gastric lesions were induced by spinal cord injury (SCI) plus diclofenac or ketorolac.

The glutathione peroxidase (GPx) levels in the sham group were approximately 1.36 nmol/min/mg protein and decreased in the SCI groups treated with ketorolac and diclofenac (Figure 4A; *p* < 0.001 and *p* < 0.002, respectively). The same trend was observed in the groups treated with methyl eugenol plus diclofenac and methyl eugenol plus ketorolac (Figure 4A; *p* < 0.003 and *p* < 0.001, respectively), as well as in the omeprazole group (reference drug). However, methyl eugenol alone maintained the enzyme concentrations without a significant difference compared to the sham group.

When total glutathione (total GSH) was measured, there was a significant decrease in the SCI group compared to the sham group (Figure 4B; *p* < 0.0005). However, the treatments with methyl eugenol, methyl eugenol plus diclofenac, methyl eugenol plus ketorolac, diclofenac, ketorolac, and omeprazole led to significant increases in total glutathione compared to the SCI and sham groups.

The levels of reduced GSH showed a decrease in all the cases except in the SCI group, with a significant difference of *p* < 0.0005 (Figure 4C).

Oxidized glutathione (GSSG) was lower in the SCI group compared to the sham group (Figure 4D), with a significant difference (*p* > 0.0005). Conversely, in the groups treated with the drugs alone or in combination with methyl eugenol (Figure 4D), the GSSG levels increased significantly compared to both the sham and SCI groups (*p* < 0.05).

### 2.3. Histological Analysis

**Figure 5:**
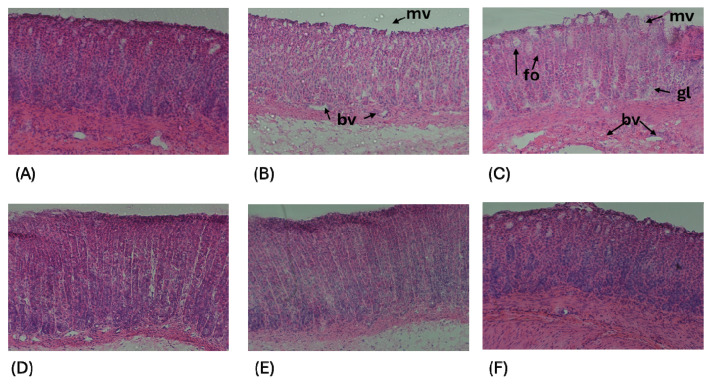
Representative images of histological sections of rat stomach, magnification at 200x. (**A**) Control group without spinal cord injury; (**B**) group treated with diclofenac; (**C**) group treated with ketorolac; (**D**) group treated with omeprazole; (**E**) group treated with diclofenac plus methyl eugenol; (**F**) group treated with ketorolac plus methyl eugenol.

The histological sections of the stomach were analyzed in the fundus, corpus, and antrum sections. In Figure 5, a representative image of the corpus of the stomach is presented. Figure 5A shows the control group with a normal morphology without visible lesions. The mucosa is observed with a simple epithelium of tall cylindrical cells that form compacted folds. In the deepest areas of the folds, gastric pits are formed. The lamina propria of the mucosa is observed without alterations; the loose connective tissue is rich in diffuse lymphoid tissue. The muscularis mucosa contains muscle fibers oriented circularly and others longitudinally.

In the group treated with diclofenac (Figure 5B), mucosal erosion with loss of continuity of the microvilli epithelium (mv), dilated blood vessels (bv) with leukocyte infiltration, and thinning of the muscularis mucosa compared to the control group (Figure 5A) were observed. In the group treated with ketorolac (Figure 5C), similar changes to those observed with diclofenac were noted, along with some open foveolae (fo) and tortuous glands (gl). In the group treated with omeprazole (Figure 5D), a decrease in mucosal erosion was observed and without opening of the foveolae or tortuosity in the glands. In the groups with the drugs diclofenac or ketorolac plus methyl eugenol (Figure 5E and Figure 5F, respectively), a decrease in damage was observed without dilation of blood vessels and a decrease in mucosal erosion.

## 3. Discussion

Traumatic spinal cord injury is a devastating condition that significantly impacts the quality of life of those individuals affected by it [13]. Traumatic spinal cord injury causes inflammation and oxidative stress locally and in organs such as the stomach due to immobilization and hypoperfusion [14]. Neuromuscular rehabilitation is usually the primary focus, but side effects such as gastric damage are less frequently explored [15]. Gastric ulcers attributed to the stress caused by permanent spinal cord injury can evolve and lead to perforation and peritonitis, potentially resulting in death [16]. In this regard, the therapeutic strategies for the treatment of peptic ulcers primarily focus on eliminating pain, reducing gastric acidity, and strengthening the gastric mucosal barrier. However, none of these strategies are completely effective, and all have side effects [17]. Therefore, there is a need for more effective and safer antiulcer agents. In the present study, the gastroprotective activity of methyl eugenol, a natural product that has already been shown to exhibit gastroprotective activity in the ethanol-induced gastric injury model [18], was evaluated in a spinal-cord-injury-induced gastric damage model in rats.

Methyl eugenol (4-allyl-1,2-dimethoxybenzene), with a molecular weight of 178 g/mol (C_11_H_14_O_2_), is an alkenylbenzene found in the essential oils of many plant species [19,20]. Alone or in combination with other components, including the structurally similar compound eugenol, methyl eugenol acts as an antioxidant [21], anti-inflammatory [22], and anesthetic [23].

This study demonstrates that oral administration of methyl eugenol exhibits dose-dependent gastroprotective activity in reducing the gastric damage caused by a traumatic spinal cord injury, which is exacerbated by the administration of diclofenac and ketorolac (Figure 1 and Figure 2). Under these conditions, gastric damage was significantly reduced by administering a dose of 177 mg/kg of methyl eugenol. This result was not significantly different from those obtained with omeprazole at a dose of 40 mg/kg, indicating that they are equally effective; however, methyl eugenol is less potent than omeprazole (Figure 1 and Figure 2).

Stress-induced gastric ulcers are known to be related to vagal hyperactivity, oxidative stress, free radical generation, and decreased gastric mucosal blood flow, all of which contribute to gastric ulcer formation [24,25]. Methyl eugenol is known to exhibit antioxidant activity through the activation of nuclear-factor-E2-related factor 2 (Nrf2). In response to oxidative stress, Nrf2 translocates into the nucleus, where it binds to conserved antioxidant response elements and activates downstream cytoprotective antioxidants such as heme oxygenase-1 (HO-1), NAD(P)H quinone dehydrogenase 1 (NQO1), and SOD [26]. This mechanism likely protects the gastric mucosa from free radicals generated by traumatic spinal cord injury [18].

Traumatic spinal cord injury causes inflammation and oxidative stress locally and in organs such as the stomach due to immobilization and hypoperfusion [14]. When measuring SOD enzyme activity, we found that it was upregulated in the traumatic spinal cord injury group and remained unchanged in the group treated with methyl eugenol. These results align with those reported by Choi et al. [27,28], who found that methyl eugenol removes radicals indirectly by upregulating the activities of antioxidant enzymes. Conversely, the application of non-steroidal anti-inflammatory drugs (NSAIDs) such as ketorolac and diclofenac, either alone or in combination with methyl eugenol, reduces SOD activity significantly (*p* < 0.0005) compared to both the sham and traumatic spinal cord injury groups. A similar effect was observed with the drug omeprazole, indicating an additive effect as ketorolac and diclofenac reduce SOD activity independently.

Additionally, when measuring the CAT enzyme, we found that it was also increased in the traumatic spinal cord injury group compared to the sham group (*p* < 0.0005). However, in the groups with SCI treated with methyl eugenol, diclofenac, ketorolac, or omeprazole, the values were similar to those in the sham group, suggesting that the upregulation of SOD effectively regulated the concentration of superoxide anion and peroxide. The resulting hydrogen is consumed through a pathway other than CAT (see Figure 3B). The additive effect of reducing the CAT concentration was observed in the groups treated with NSAIDs plus methyl eugenol (methyl eugenol plus diclofenac or ketorolac).

Another mechanism to regulate hydrogen peroxide, distinct from the CAT enzyme, is GPx, which clearly coincides with the CAT and GPx data. The SCI group showed increased CAT and decreased GPx compared to the respective sham group. In the group treated with methyl eugenol, no significant difference was found in the concentrations of CAT and GPx compared to the sham group; however, there was a significant difference when compared with the respective SCI group. This indicates that the spinal cord injury itself is the stimulus for the changes in the CAT and GPx concentrations. Furthermore, when the anti-inflammatory drugs ketorolac and diclofenac are combined with methyl eugenol in the spinal cord injury model, both the CAT and GPx concentrations decrease compared to the sham group. However, when ketorolac, diclofenac, or omeprazole are administered regarding the spinal cord injury model, GPx decreases, but the CAT levels remain unchanged compared to the sham group. This indicates that methyl eugenol maintains an antioxidant protective effect through both CAT and GPx increases.

The results of the total GSH determination align with the findings reported by Zheng [29], which indicate that some NSAIDs, such as diclofenac, can increase total GSH concentrations. When investigating whether this increase is due to reduced glutathione (GSH) or oxidized glutathione (GSSG), we found that total GSH was increased at the expense of GSSG, indicating the activation and response of the GSH antioxidant pathway [30].

Regarding MDA, it is one of the most used markers for lipid peroxidation. MDA is generated in vivo through the peroxidation of polyunsaturated fatty acids. The result in the sham group could be considered a baseline when performing the intervention. Surprisingly, in the SCI group, the MDA levels were significantly lower (*p* < 0.004) compared to the sham group, which may be related to gastric hypoperfusion [31] caused by spinal cord injury and the timing of determination (18 h post-injury). Conversely, in the group treated with methyl eugenol, the MDA levels significantly increased (*p* < 0.0005), similar to the observations in cases of ischemia-reperfusion [32]. This effect is diminished when NSAIDs like ketorolac and diclofenac are added, which alone reduce MDA concentrations to less than 2.0 × 10^−7^ mmol/µL/mg protein.

The macroscopic analysis coincides with the microscopic one in the control group that we observed without a blood band and intact mucose, respectively. The same occurs in the groups with diclofenac and ketorolac in the macroscopic analysis, which observed hemorrhagic bands, and in the microscopic analysis, in which mucosal erosion was found that coincided with the report by Tandoh et al., 2021 [33]. However, when the groups were treated with methyl eugenol, the damage was diminished in both analyses.

## 4. Materials and Methods

This study was approved by the Ethics, Bioethics, and Biosafety Committee of the Facultad de Estudios Superiores Zaragoza, UNAM, under registration FESZ-CE/21-118-18.

### 4.1. Drugs

Methyl eugenol was suspended in a 0.05% carboxymethylcellulose solution (vehicle 1). Water (vehicle 2) was used to dissolve diclofenac sodium, ketorolac tromethamine, and omeprazole. All drugs were purchased from Sigma Chemical Co. (St. Louis, MO, USA), and all substances were prepared immediately before use. The assay kits were acquired from Cayman Chemical Co. (Ann Arbor, MI, USA) and Bioassay Systems (Hayward, CA, USA).

### 4.2. Animals

Wistar rats (200–250 g) from the Facultad de Estudios Superiores Zaragoza, UNAM, Mexico City, Mexico, were used in this study. They were provided free access to water throughout the procedures. All experiments were conducted in accordance with the Official Mexican Standard for the care and use of laboratory animals (NOM-062-ZOO-1999).

### 4.3. Transection of the Spinal Cord

The rats were anesthetized with an intramuscular injection of ketamine (80 mg/kg) and xylazine hydrochloride (8 mg/kg) [34]. After verifying the induction of anesthesia, an aseptic laminectomy was performed at the T9 level [34]. The spinal cord was transected by sliding an 11-gauge scalpel blade through the dura mater. The accuracy of the lesion was confirmed through visual inspection and by passing a microhook along the dura mater’s internal contour [35]. The soft tissues were sutured in layers. For the sham injury, anesthetized animals underwent soft tissue surgery only [35]. After anesthesia recovery, the animals were housed individually in cages with wire-net floors. They were deprived of food for 18 h before experimentation, with unrestricted access to water throughout.

### 4.4. Evaluation of the Gastroprotective Activity of Methyl Eugenol

To assess gastric damage resulting from diclofenac or ketorolac, eight rats were used per group. All animals underwent spinal cord injury. Group 1 was the vehicle control group and received vehicle 1 (0.5 mL/100 g, orally). Group 2 (diclofenac control group) also received vehicle 1, while groups 3 to 5 received methyl eugenol (30, 100, and 177 mg/kg, respectively) by the same route and volume. Group 6 received omeprazole (40 mg/kg) [36]. Thirty minutes later, group 1 was administered vehicle 2, while groups 2 to 6 received diclofenac (30 mg/kg). Six hours later, the animals were sacrificed in a CO_2_ chamber, and their stomachs were dissected and filled with 2% formalin. After five minutes, the stomachs were opened along the greater curvature, and the lesion area was measured in mm^2^, constituting the ulcer index. The same methodology was followed for ketorolac (30 mg/kg) -induced gastric damage.

The lesion area was determined blindly using a stereoscopic microscope with a metric grid. The total area of all lesions in each stomach represented the ulcer index [37].

### 4.5. Determination of Antioxidant Activity

For this evaluation, the dose of 177 mg/kg of methyl eugenol was chosen as it provided the greatest gastroprotection. Group 1 was a sham group, and groups 2 to 8 had spinal cord injuries and were treated as shown in Table 1. The methodology was similar to that used in the gastroprotection evaluation, with modifications described below.

After sacrificing the animals, the stomachs were washed with PBS buffer (pH 7.4) to remove red blood cells or clots. The body of each stomach was scraped and homogenized 20 times for periods of 1 min in 5 mL of a homogenizing solution (containing 250 mM sucrose, 2 mM MgCl_2_, 1 mM EGTA, and 2 mM Hepes, pH 7.4, with 0.1% protease inhibitor cocktail) at 6000 rpm using a tissue homogenizer on an ice bath [38]. The homogenate was centrifuged at 6000 rpm for 30 min at 4 °C. The supernatant was aliquoted and frozen at −80 °C until the antioxidant activity determinations following the manufacturer’s instructions [39].

Superoxide dismutase (SOD) activity was determined using an assay kit (Cat # 706002, Cayman Chemical Co.). The results were expressed as units (U) of SOD activity/mg of protein. The decomposition of hydrogen peroxide (H_2_O_2_) in the presence of the enzyme catalase was estimated according to the assay kit (Cat #. 707002, Cayman Chemical Co.). The results obtained are expressed in nmol/min/mg of protein. Malondialdehyde (MDA) content was quantified using an assay kit (Cat #. 10009055, Cayman Chemical Co., Ann Arbor, MI, USA); the levels were expressed as mmol/μL/mg protein.

The glutathione peroxidase (GPx) activity was determined in nmol/min/mg of protein according to the manufacturer’s specifications (Cat #.703102, Cayman Chemical Co.). Finally, the total GSH, GSH, and GSSG levels were estimated following the manufacturer’s instructions (BioAssay System Cat #. EGTT-100). The results were expressed as μM. The absorbance of all results was measured spectrophotometrically at different wavelengths corresponding to each test according to the manufacturer’s specifications [40].

### 4.6. Histology

The stomachs of the rats were divided into the fundus, body, and antrum and cut into fragments of approximately 0.5 × 0.5 cm. These fragments were immediately placed in optimal cutting temperature compound (OCT or Tissue-Tek) (Leica, Nussloch, Germany), frozen with liquid nitrogen, and stored at −80 °C until histological sectioning. Sections were cut at 4 microns using a Cryostat (Leica, model CM100-3) and placed on glass slides. The slides were then stained with hematoxylin for 10 min and eosin for 1 min. The histological sections were examined under a microscope to observe the integrity of the gastric mucosa, submucosa, muscularis externa, and serosa.

### 4.7. Statistical Analysis

The statistical significance between treatments was examined by one–way analysis of variance (ANOVA) followed by Dunnett’s test when the data presented a normal distribution. In non-parametric data, the Kruskal–Wallis test followed Dunn’s test. Data are expressed as the mean ± SEM (*n* = 8), with significance considered at *p* ≤ 0.05.

## 5. Conclusions

Patients with traumatic spinal cord injury are highly susceptible to developing gastric ulcers, which severely impact their quality of life and can lead to life-threatening complications. This study suggests that methyl eugenol may offer a promising alternative for managing this condition. Our findings demonstrate that methyl eugenol provides significant gastroprotective effects at both the macroscopic and microscopic levels against SCI-induced gastric damage, particularly in the presence of diclofenac or ketorolac. These protective effects are mediated, at least in part, by the enzymatic activities of glutathione peroxidase and catalase.

## Figures and Tables

**Table 1:** Experimental design for the evaluation of antioxidant activity of methyl eugenol (177 mg/kg) on gastric damage in SCI rats with oral administration of diclofenac (30 mg/kg) or ketorolac (30 mg/kg).

Group	Spinal Cord Injury	Treatment	Gastric Damage
1	------	Vehicle 1	Vehicle 2
2	✓	Vehicle 1	Vehicle 2
3	✓	Methyl eugenol	------
4	✓	Methyl eugenol	Ketorolac
5	✓	Methyl eugenol	Diclofenac
6	✓	Carboxymethylcellulose	Ketorolac
7	✓	Carboxymethylcellulose	Diclofenac
8	✓	Omeprazole	------

Vehicle 1: carboxymethylcellulose; vehicle 2: water. The ✓ symbol means it has the spinal cord injury.

## Data Availability

The data presented in this study are available on request from the corresponding author.
